# Profound Love and Dialogue: Paulo Freire and Liberation Education

**DOI:** 10.3928/24748307-20240613-02

**Published:** 2024-07

**Authors:** Darriel B. Harris, Debra L. Roter

## Abstract

Paulo Freire, Brazilian philosopher and educator, proposed an educational method for adults based on what he called “the problem posing method.” This method was based on dialogue that he deemed necessary for education and was not oppressive and controlling. Freire argued that traditional educational methods were inherently oppressive because they served the interest of the elite, instituted what he called “the banking method” in hopes to turn people into better workers. In contrast to this, Freire advocated for an education that was liberating and required dialogue. Dialogue, however, could only take place with profound love. This article reflects on Freire's call for profound love and dialogue within his pedagogical framework, and its necessity for social and political change. Further, this article explores what Freire meant by profound love and dialogue, and explores how love and dialogue are applicable to current and future health literacy and health education efforts. [***HLRP: Health Literacy Research and Practice*. 2024;8(3):e118–e120.**]

Paulo Freire was a Brazilian philosopher and educator known worldwide for his method of education as a vehicle of liberation and social change through individual and community empowerment (Freire, 1973). Fundamental to Freire's educational method is that equal participatory dialogue leads to independent actions. Freire describes strategies for engaging learners in participatory dialogue yet notes that these educational strategies only lead to the learner's liberation when conducted with humility and profound love (Freire, 2000). Freire (2000) understands love to be so critical to his philosophy and method that he writes, “Dialogue cannot exist, however, in the absence of a profound love for the world and for people” (Freire, 2000, p. 89). The purpose of this Brief Report is to reflect on what is meant by profound love in Freire's work and how and why it is necessary for empowerment and social change.

## An Historical Perspective on Adult Education

As a point of perspective, Freire was not the only thought leader to prescribe educational methods that treated adult learners as equal to their teachers. Malcolm Knowles, who led the first organized adult education efforts in the post-World War II America, developed the theory of andragogy. Knowles defined andragogy as “education of equals,” which was distinguished from traditional “education from above” methods of pedagogy used in the education of children (Knowles, 1980).

Knowles presented andragogy as the pathway to successful adult education based on the psychology of adult learners noting that adults (unlike children) are internally motivated by life experience to determine what information is relevant and applicable to problems they want to address (Knowles, 1980). While the work of both Knowles and Freire recognized many of the same failures of traditional education to meet the needs and build upon the strengths of adult learners, they did so with different intent. In his autobiography, Knowles (1989) concludes: “I am just not good at political action. My strength lies in creating opportunities for helping individuals become more proficient practitioners” (p. 146). This is the crux of the difference between Knowles and Freire.

Freire, like Knowles, regards education of equals as a foundational element of adult education, but he differs in framing the purpose of education to be individual and community liberation, political or otherwise (Freire, 2000). Freire's educational philosophy grows out of his commitment to humanism. Freire begins his most forceful writing, *Pedagogy of the Oppressed*, by noting that humanistic concerns lead to the realization of the historical and present gravity of dehumanization, particularly as it foisted on the less powerful segments of society (Freire, 2000). Freire (2000) goes on to note that dehumanization—“injustice, exploitation, oppression, and the violence of the oppressors”—at its core is the loss of one's ability to name their experiences and the environment in which they live, being dependent on others' naming and interpretations (p. 44). Further, Freire (2000) writes, “To exist, humanly is to name the world, to change it” (p. 88). Education, for Freire, is a journey toward becoming more human, a journey toward naming one's world, recognizing the oppressive and dehumanizing elements in one's life experience and transforming them to elements of freedom and justice (Freire, 2000).

To achieve liberation from oppression, Freire calls for an end to “the banking method of education” in which the learner is viewed as an object into which the teacher deposits knowledge and from whom little competence, creativity, or cognitive processing is expected (Freire, 2000; Freire, 1973). In the banking method, even if the student regards the information relevant, the learners' world and experience is named by others and thus inherently remains oppressive (Freire, 2000). Instead of banking, Freire (2000) calls for problem-posing education, where those present are teacher-students and student-teachers, wherein “the teacher presents the material to the students for their consideration and reconsiders earlier considerations as the students express their own” (p. 81). Problem-posing is always a cognitive process that “involves a constant unveiling of reality…[and] strives for the emergence of consciousness and critical intervention in reality” (Freire, 2000, p. 81).

## What Does Love Have to do With It?

Freire's emphasis on reality requires the Freirean teacher to acknowledge and affirm the reality of the student. To affirm a student's reality requires the teacher to learn the student's reality and regard it with import and meaning (Freire 2000). This is a loving act which traditional education cannot perform because it fails to center on the student's liberation but instead centers on making informational deposits. Consequently, Freire believed that the foremost duty of a teacher is to create and maintain a dialogical exchange with students, writing “love is at the same time the foundation of dialogue and dialogue itself” (Freire, 2000, p. 89). Through dialogue and an emphasis on student's reality, the teacher participates in the student's humanistic growth, becomes the student's ally, and helps the student name, critique, and alter the world they inhabit (Freire, 2000).

Freire believed that it is profound love that centers the students' liberation (Freire, 2000). Without profound love, the teacher lacks the courage and motivation to challenge the status quo, address oppressive educational elements, and dismiss notions of expert superiority (Freire, 2000). Friere notes, “love is an act of courage, not of fear” (Freire, 2000, p. 89). Whereas fear seeks to control another, love seeks to see another blossom, both the world and the student, in all possible forms (Freire, 2000). Profound love also enables the teacher to recognize that the humanity of the student is equal in value to their own humanity (Freire, 2000). Thus, to operate in a manner that affirms equal humanity is not a strategy nor a means to educate, but a foundational truth upon which education can take place (Freire, 2000).

Freire believed that love is also a requirement of the student (Freire, 2000). Without love of self and others, students will deny their own perspective, view themselves as inferior, and be over-reliant on the perspective of the teacher (Freire, 2000). With the presence of love, including love of self, the student can find value in their own experiences and regard the teacher as an equal human with different experiences with whom to discuss perspectives (Freire, 2000). In this way, the student and the teacher grow further into their respective humanities to the benefit of each other and the worlds they inhabit. Freire (2000) notes, “the great humanistic and historical task of the oppressed [is to] liberate themselves and their oppressors as well” (p. 44).

## The Freirean Legacy for Teacher Training and Health Education

Freire's legacy is evident in processes that no longer view education simply as a skill building initiative, or an information transfer, but understand education as a liberating quest toward the betterment of individuals and their place in collective humanity. Because it is loving, it desires the best for the student and stands in opposition to whatever hinders human expression while insisting that all humans be expressive.

Speeches, sermons, lectures, informational videos, and other forms of educational monologue can inspire or even motivate, but they cannot provide the opportunity for a critical and conscious reflection of life experiences. These are useful only as a starting place. The learner as listener is unable to frame the information conveyed, name the assumptions upon which it stands, nor determine the vocabulary and language used to define its context and consequences. Authentic dialogue is the alternative to monologue. It requires the establishment of a relationship through which participants truly see and recognize the personhood of the other and the worlds they inhabit.

Love is a difficult and unusual directive to follow. Educators schooled in Freirean methods are trained to use learning strategies and techniques that are effectively matched to student needs and strengths, and to elicit and engage in participatory dialogue. Training manuals do not provide instruction or suggest strategies to achieve relationships of “profound love'' yet it is crucial to Freire's educational philosophy in that only profound love is powerful enough to motivate the abandonment of the power and prestige of expertness necessary for authentic dialogue.

Freire's legacy is also reflected in community based participatory research (Wallerstein et al., 2020) and community-engaged research (Ortiz et al., 2020) initiatives designed to reduce health disparities. It is also reflected in the broadening definition of medical care quality to include patient-centered communication (Roter, 2000) and care that prioritizes the inclusion of patient voice in setting medical goals, directing management and treatment priorities, and defining quality of life and well-being (Gerteis et al., 2002). Freire's influence has also shaped the field of Health Education generally (Bartlett, 1986; Roter et al., 2001; Wallerstein et al., 1988; Wallerstein et al., 1994), through participatory production of health education materials such as photo novellas (McGinnis et al., 2014) and photovoice (Strack, 2022). Finally, Freire has inspired engagement of faith-based communities in the production and dissemination of health behavior messages and materials (Harris, 2021).

The commitment of health education to Freirean principles and the empowerment agenda adopted some 50 years ago challenges the profession not only to serve community educational needs but to empower communities to define their own needs and challenge institutions to serve those community defined needs. It will take profound love, dialogue, and courage for this work of liberation to continue and succeed.
